# Successful Treatment of Hyaluronic Acid-Induced Facial Necrosis With Intravenous Dinoprostone

**DOI:** 10.7759/cureus.68847

**Published:** 2024-09-07

**Authors:** Olivia Gaudissart, Axel De Greef, Hugues Fierens, Marie Baeck

**Affiliations:** 1 Department of Dermatology, Cliniques universitaires Saint-Luc (UCLouvain), Brussels, BEL; 2 Department of Dermatology, Clinique Saint-Jean, Brussels, BEL

**Keywords:** complication, dinoprostone, fillers, hyaluronic-acid, skin necrosis

## Abstract

There is an increasing interest in the use of facial dermal filler injections, such as hyaluronic acid, for esthetic and rejuvenation purposes. However, their use is associated with a potential risk of complications.

We report the case of a woman in her thirties, with rapidly evolving facial skin necrosis following accidental intravascular injection of hyaluronic acid and/or extravascular compression. The necrosis worsened despite early repeated local injections of hyaluronidase. However, rapid and favorable clinical evolution was observed after the patient was treated for seven consecutive days with intravenous infusion of dinoprostone (a prostaglandin E2).

## Introduction

Interest in esthetic medicine is growing in dermatology practice. Some procedures involve minimally invasive non-surgical rejuvenation techniques such as hyaluronic acid (HA) injections for soft tissue filling or contouring. The common mild and temporary complications include ecchymosis, pigmentation, granuloma formation, and hypersensitivity reactions, but it is not uncommon to encounter more severe and sometimes irreversible complications, such as vascular issues that may lead to skin necrosis and distal complications like blindness or stroke. The lack of a framework for the training of aesthetic procedures increases the risks of those side effects and complications [1], the incidence of which is likely to be underreported and thus underestimated. Cutaneous necrosis is one of the most serious complications induced by accidental intravascular injection of HA. To date, immediate and high-dose pulsed hyaluronidase injections are considered the gold standard to treat dermal necrosis, but with inconstant results [2]. Dinoprostone was used in the present case to increase blood flow in the affected area and seems to be a safe second-intention therapy after the failure of local injections of hyaluronidase.

## Case presentation

An otherwise healthy Mediterranean woman in her 30s was referred to the emergency dermatology consultation of our tertiary hospital for treatment of facial skin necrosis three days after receiving HA injections to her right nasal fold. Painless and instant skin blanching was observed during the needle injection procedure and prompted immediate local injections of hyaluronidase (450 IU). The lack of improvement and the appearance of cyanosis motivated repeated injections of hyaluronidase (1500 IU each day, over the following three days). Despite well-administered local treatment and the addition of methylprednisolone 32 mg/day and aspirin 100 mg/day within the following 24 hours, the clinical aspect worsened.

**Figure 1:**
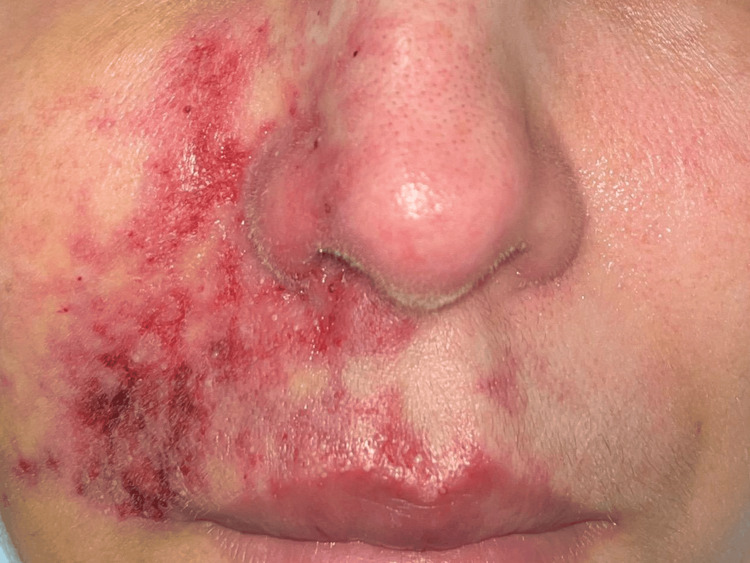
Clinical features of the patient at admission We observed a large erythematous livedoid area on the right nasal fold and cheek with a few pustules and with the initial stage of cutaneous necrosis on the upper lip.

At admission to our department, four days after the HA injection, we observed a large erythematous livedoid area on the right nasal fold and cheek with a few pustules, and with initial stage of cutaneous necrosis on the upper lip (Figure 1). Except for local pain, she denied any systemic symptoms or fever. Laboratory results showed an increased C-reactive protein level (38 mg/L; normal value (NV) <5 mg/L) and grade II lymphopenia (610/µL; NV: > 1500/microL), but liver and kidney functions, as well as coagulation factors, were normal.

Given the failure of previous therapies, we administrated intravenous continuous dinoprostone (a prostaglandin E2 (PGE2)) at a dose of 0.75 mg per day for 7 consecutive days during her hospitalization. Oral methylprednisolone (32 mg/day) and oral aspirin were continued. Local treatment consisted only of daily disinfection (chlorhexidine) and paraffin gaze (Jelonet®).

Clinical improvement was observed 24 hours after the start of the PGE2 infusion and continued up to day seven. The treatment was well tolerated, apart from mild diarrheas and moderate hypokalaemia (3.16 mmol/L; NV: 3.5-4.5 mmol/L) (Table 1), probably due to systemic glucocorticoids and digestive losses but rapidly corrected with oral potassium gluconate supplementation.

**Table 1:** Laboratory investigations

Test	Result	Reference range
C reactive protein	38 mg/L	< 5 mg/L
Lymphocytes	160 µ/L	> 1500 µ/L
Serum potassium	3,16 mmol/L	3.5–4.5 mmol/L

**Figure 2:**
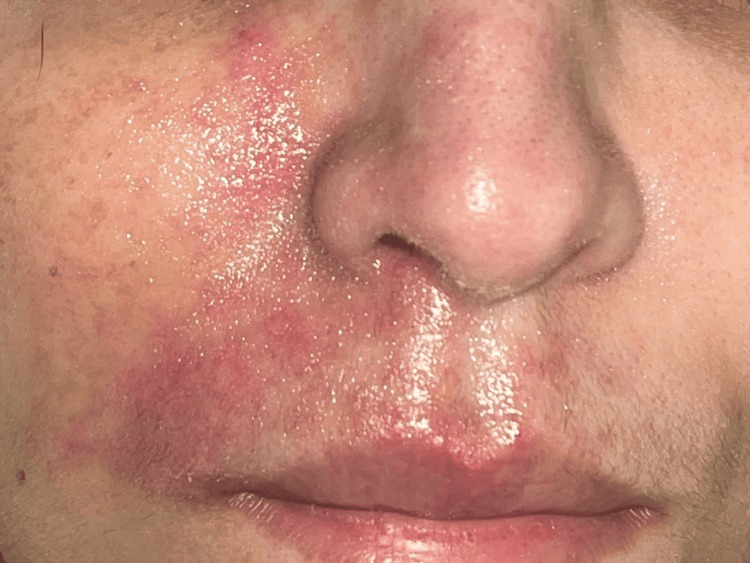
Clinical evolution after seven days of continuous intravenous dinoprostone We noticed a few post-inflammatory marks but no skin defect and no cutaneous necrosis.

One week later, at the time of hospital discharge, healing was nearly complete, with no skin defects other than a few post-inflammatory marks (Figure 2).

**Figure 3:**
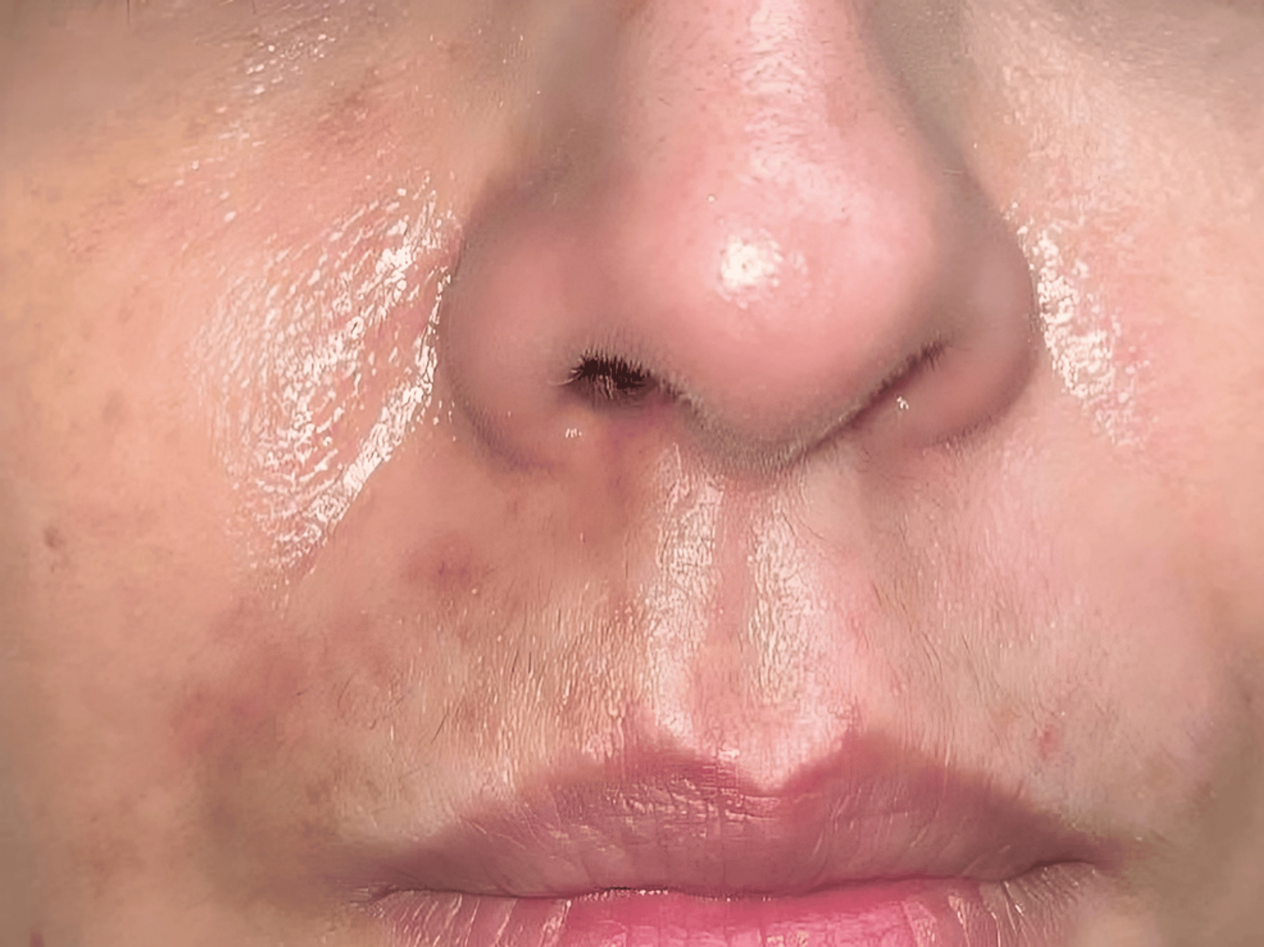
Clinical evolution one month after intravenous continuous dinoprostone Skin healing is complete, we noticed some light post-inflammatory marks.

At the follow-up visit one month later, healing was maintained and complete (Figure 3).

## Discussion

Our patient presented with necrosis of the distal territory of her right facial artery probably secondary to thrombosis and occlusion by accidental intra-arterial injection of HA.

Skin necrosis following accidental arterial injection of HA is rare (estimated at 0.01% of all filling procedures performed [3]) but represents a serious complication that can lead to tissue loss and significant scarring if not treated appropriately and rapidly. The use of a flexible cannula instead of a sharp needle to inject HA seems to reduce the risk of such complications [4].

Symptoms usually occur immediately or a few hours after the injection. The clinical condition is called Nicolau syndrome and is characterized by immediate pain with pallor and/or livedoid aspect, followed by erythema, edema, pustules, and necrosis of the regional skin area [5].

Current management guidelines based on case reports and patient series recommend injections of hyaluronidase in the area of the necrosis (minimum 1500UI, within the first 72 hours of the first symptoms of vascular compromise) [6]. Hyaluronidase can dissolve HA but is not Food and Drug Administration (FDA)-approved for this specific indication and, therefore, its use in treating necrosis is considered off-label [7]. Moreover, it is important to consider that local injections of hyaluronidase may add further trauma to the initial lesions with the risk of complete lysis of the residual extracellular matrix already weakened in the compromised area. These multiple micro-traumatic injections also increase the risk of introducing microorganisms into the ischaemic and necrotic area leading to cutaneous infections.

Other treatments have been suggested, such as massages with topical nitroglycerin, aspirin, low molecular weight heparin (due to its anticoagulant action and anti-inflammatory properties), as well as sildenafil [8].

Moreover, systemic vasodilatory treatments, aimed at encouraging vascular recovery of the compromised territory by increasing peripheral shunts, can help limit the extent of the necrosis. Prostaglandin E2s, like dinoprostone, whose vasodilatory properties have been shown to be effective in peripheral vascular diseases or filler secondary embolisms, have been successfully used to treat skin necrosis following HA intra-vascular injections [9]. However, they are not included in the recent treatment algorithm for HA-related complications [10]. In recent years, increasing evidence has shown that prostaglandin E2 (PGE2), may potentiate tissue regeneration and repair following injury in diverse organ systems including the skin [11]. All fillers can induce skin ischemia and the pathogenesis of necrosis induced by HA injection is not well understood. Several factors are involved, and the final skin lesion can be due to a combination of angiospasm, inflammation of the arteries, and thrombotic occlusion of the arterioles [12]. As said, dinoprostone was used in the present case to increase blood flow in the affected area.

## Conclusions

There is an increasing interest in the use of facial dermal filler injections, such as hyaluronic acid, for soft tissue filling or contouring. However, their use is associated with a potential risk of complications. Cutaneous necrosis is one of the most serious complications induced by accidental intravascular injection of HA and/or extravascular compression.

Current guidelines recommend immediate injections of hyaluronidase in the area of the necrosis. However, the results of such treatment are not constant. Intravenous injection of prostaglandin E2 (PGE2) seems to be an effective and well-tolerated alternative treatment.
